# Functional Diagnosis of Kidney Disease

**Published:** 1904-01

**Authors:** 


Functional Diagnosis of Kidney Disease. With especial reference to Renal Surgery. Clinical experimental investigations by Drs. Leopold Casper and Paul Friederich Richter, Berlin. Translated by Dr. Robert C. Bryan, Richmond, Va., and Henry L. Sanford, Cleveland, O. Duodecimo, pages 233. Philadelphia: P. Blakiston's Son & Co. 1903. (Price, $1.50.)

The first thing which strikes even a casual observer on opening this book is its apparent completeness, and its value is increased by the reports of cases which it contains illustrative of the body of the work. Certainly there are no more expert workers in the renal field than Casper and Richter, and they have given the results of their research, their observations, and their deductions to the profession with all the detail which marks the literary efforts of the Germans. The translators have given the book a rather free rendering and have not marred it by attempting to add a great mass of personal opinion and American references. They have shown their wisdom in being content to translate the work of the eminent foreigners and give the words of Casper nearly as they were originally published. Moreover, in the translation nothing has been lost, the work being very complete. There is much space given to ureteral catheterisation, its difficulties, its advantages and its errors. The problems of diagnosis, the importance of functional diagnosis and its methods are fully described, as is the determination of urinary constituents. Cryoscopy is explained intelligently and in a manner simple and sensible. A large part of the book is given up to illustrative case reports and experimental results. The authors have not written against space, but have made every word count, which is another reason why they have given us such a valuable book. N. W. W.

